# Establishing UK research priorities in smell and taste disorders: A James Lind alliance priority setting partnership

**DOI:** 10.1111/coa.13985

**Published:** 2022-10-02

**Authors:** Carl M. Philpott, Andreas Espehana, Mairenn Garden, Ngan Ta, Nishita Gadi, Kala Kumaresan, Devina Maru, Lorenzo D. Stafford, Nina Bleasdale, Duncan Boak

**Affiliations:** ^1^ Norwich Medical School University of East Anglia Norwich UK; ^2^ The Norfolk Smell & Taste Clinic Norfolk & Waveney ENT Service Norwich UK; ^3^ Fifth Sense Barrow‐in‐Furness UK; ^4^ ENT Department Norfolk and Norwich University Hosptial NHS Trust Norwich UK; ^5^ Department of Medicine Anglia Ruskin Univeristy Chelmsford UK; ^6^ National Clinical Champion for Deafness and Hearing Loss Royal College of General Practitioners London UK; ^7^ Department of Psychology University of Portsmouth Portsmouth UK

**Keywords:** advocacy, anosmia, hyposmia, olfactory disorders, parosmia, phantosmia

## Abstract

**Objectives:**

To determine the top 10 research priorities in Smell and Taste Disorders (SATD).

**Design:**

After steering group was established, an electronic survey was disseminated to determine the list of questions. After removing out‐of‐scope responses, the remainder were consolidated to create summary questions. A literature search was conducted to remove already answered questions. A second survey was used to determine the top questions that formed the subject of final debate at a workshop attended by clinicians and patients to determine the top 10 priorities.

**Setting:**

A James Lind Alliance Priority Setting Partnership (JLAPSP) was established by FifthSense to identify the top 10 research questions in SATDs in the United Kingdom.

**Participant:**

All stakeholders in SATDs (patients, healthcare professionals, family, carers, researchers).

**Main outcome measures:**

Final 10 research priorities.

**Results:**

The 665 respondents to the initial survey provided 1698 research questions. Thirteen were out‐of‐scope and removed; remaining 1685 were then consolidated to form 147 summary questions. Following literature search and discussion with the steering group, 37 questions remained for the second survey, which 235 people responded. The top ten priorities agreed upon in the workshop covered themes of improved understanding of pathophysiologlogy, improving health services, and managing long‐term effects of smell/taste disorders. The most important research question agreed was “How can we further our understanding of the mechanism of disease in the nerve pathways that affect smell and taste disorders, including where parosmia and phantosmia exist.”

**Conclusion:**

We report the top 10 research priorities in smell and taste disorders. These priorities will now empower researchers to secure research funding and provide the basis of the FifthSense research hub.

**Key Points**

1. The aim of the PSP was to identify unanswered questions about smell/taste disorders from all stake holders in the UK, and agree which are the most important for research to address.
2. The scope included questions about diagnosis, understanding pathophysiology, treatment (new drug trials/devices), and access to care for smell and taste disorders. Questions raised about non‐UK care/treatment and normal variations of smell and taste were regarded out of scope.
3. While the respondents of our survey consisted of a largely white, older female demographic, this was considered during discussion in the final workshop and discussions ensured balance in the final top 10 research priorities.
4. Various stakeholders were invited to take part in the process including other charities in the field and professional bodies representing clinicians.
5. The results of this PSP should be used to guide research in smell and taste disorders in future for both researchers and research commissioners.

## 1 INTRODUCTION

Smell and taste disorders (SATDs) feature symptoms of anosmia (total smell loss), parosmia (smell distortion), phantosmia (smell hallucination), and dysgeusia (taste disturbance). Studies in other European countries show varying prevalence, depending on the methods undertaken; persistent symptoms of anosmia affect 1%–5% of the population, while hyposmia and other smell disturbances affect about 20%, increasing over the age of 60.^1^, ^2^, ^3^ These European estimates^4^ suggest anosmia is more prevalent than profound hearing loss or blindness in the UK. True taste loss is rare, but 60% of SATD patients report loss of flavour. In terms of gender differences, research shows that men and women are not impacted equally from SATDs; men appear worse affected than women when smell tests are used^4^, ^10^ and yet women present with SATDs more readily.^7^

SATD causes include sinonasal disease (62%) and post‐infectious olfactory dysfunction (PIOD) (11%), with other causes including head trauma and idiopathic cases where symptoms are more likely to be persistent.^8^ Changing prevalence is now occurring with PIOD resulting from the global SARS‐CoV‐2 pandemic; >60%–75% of those infected experience anosmia^9^, ^10^ (>150 million people globally). Although the majority recover within 4 weeks of the onset, current data suggests that 10% have continued SATDs without spontaneous recovery.^11^

Recent evidence from several population studies show that anosmia is an independent risk factor for shortened life span,^5^, ^6^ with anosmia acting as a marker of cumulative toxic environmental exposures. Furthermore, there are far reaching daily impacts on quality of life.^7^ SATD patients report high rates of depression (49%), anxiety (47%), impairment of eating (95%), isolation (64%), and relationship difficulties (59%), creating a long‐term psychological burden in sufferers, in addition to their risk of decreased lifespan.^13^, ^14^ However, patients often feel that they have not been well‐managed and their condition is trivialised.^12^, ^15^ Our recent study (*n* = 673) showed wide variation in clinical practice and little consistent information provided by clinicians to patients on prognosis or treatment. SATD patients struggle to get access to the right care and support for their condition; 76% reported their condition was recognised by an otorhinolaryngologist, 64% by GPs, and 47% by neurologists.^16^ There is a clear evidence gap for treatments^17^ and thus clinicians' interest wanes leaving patients without therapy or empathy.^16^

FifthSense is the charity for people affected by SATDs, established to transform the way in which the disorders are understood, treated, and managed. As part of this initiative, FifthSense has worked in partnership with the University of East Anglia and under the guidance of the James Lind Alliance to determine future research aims. There is a documented mismatch between research which is undertaken and the priorities of patients and healthcare professionals.^18^ The James Lind Alliance (JLA) is a non‐profit initiative, which aims to identify and prioritise unanswered research questions through bringing together patients, carers, and clinicians in Priority Setting Partnerships (PSP) to determine what they agree is most important.

The overarching aim of the PSP was to identify the unanswered questions about smell/taste loss/disturbances from patient, carer, and clinical perspectives and then prioritise those that patients, carers, and clinicians in the UK agree are the most important for research to address.

The objectives of the PSP were to:

- work with patients, carers, and clinicians to identify uncertainties about the causes and treatments of smell loss and access to care by patients
- agree by consensus a prioritised list of those uncertainties, for research
- publicise the results of the PSP and process
- take the results to research commissioning bodies to be considered for funding.

## 2 METHODS

The methods described here followed a set process predetermined by the James Lind Alliance (JLA Guidebook | James Lind Alliance (nihr.ac.uk)). The teams involved were guided throughout by the presence of a JLA advisor.

The reporting guideline for priority setting of health research (REPRISE) was used.

### 2.1 Steering group formation

The steering group consisted of a group of stakeholders representing clinical, patient, and research domains and included ENT specialists, a neurologist, a general practitioner, psychologist, and six patient representatives covering the main causation groups for SATDs. A JLA advisor was present to assist the steering group as a neutral representative to ensure that the entire process followed protocol. Members of the steering group joined on a voluntary basis. The steering group was responsible for discussing what implications the scope of the PSP had for the evidence‐checking stage of the process.

### 2.2 Scope of the PSP

The scope for the PSP was discussed and decided upon by the steering group. Questions raised by respondents of the initial survey asking about diagnosis, understanding pathophysiology, treatment (new drug trials/devices), and access to care will be regarded as in scope. As this is a UK‐based study, uncertainties raised by respondents about non‐UK care/treatment and normal variations of smell and taste will be regarded as out of scope.

### 2.3 Initial survey and identification of themes

The initial survey was sent out in late 2020 asking for respondents to state what their research priorities are regarding SATDs. Each respondent could submit up to three uncertainties as a free text format. Respondents of the survey were also asked to provide demographic details. Respondents identify as someone who suffers from SATDs, someone who cares for a person with a SATD, a healthcare professional, or a researcher in the field. If they identified as someone who suffers from a SATD, they could select which symptoms they experience, whether a cause has been identified and whether they have had access to treatment.

The target audience of the survey included, patients, clinicians/scientists/researchers, other professionals (third sector, journalist, educators, corporate), families, carers, and anyone with a general interest. To reach as wide an audience as possible, multiple avenues were utilised to disseminate the survey. The survey was published on the FifthSense website along with a press release. Commercial partners of FifthSense were also approached to help spread the survey, and these include Cadent and FlavorActiv. Professional partners including the British Rhinological Society, ENT UK and the Global Consortium for Chemosensory Research were also engaged to send out the survey to their members. A full list of organisations and institutions approached to help to disseminate the survey can be found in Table 1.

**TABLE 1:** List of organisations approached to disseminate initial survey

AbScent Anosmie.org AgeUK Parkinsons Alzheimers UK UK Acquired Brain Injury Forum The Neuroalliance British Rhinology Society ENTUK Headway Mind Life Kitchen UK Semio‐chemisty Network European Rhinology Society European Chemoreception Research Organisation University Florida Centre for Smell and Taste Tufts University Rocky Mountain (Canada) Clinical Olfactory Working Group (COWoG) AChemS International Symposium for Olfactory and Taste (ISOT) Monell Global Consortium for Chemosensory Research (GCCR) Royal College of Surgeons‐ England Royal College of Surgeons‐ Edinburgh Royal College of Physicians and Surgeons of Glasgow Royal College of Surgeons Ireland Royal College of Psychiatry Royal College of Physicians Neurology group British Psychological Society ResearchGate ARS AAOHNS

### 2.4 Indicative questions and evidence search

A team of data analysts was recruited to help process the information provided by the survey. All of the data analysts were junior doctors or medical students. All work undertaken by the data analysts were reviewed by the steering group and independent JLA advisor. All the ‘raw’ uncertainties (research questions) raised in the initial survey were grouped into themes identified by the data analysts and all uncertainties that were out of scope were removed with their respective reasons identified. All out of scope uncertainties have also been reviewed by the steering group to ensure that they were truly out of scope.

In each of the themes, the ‘raw’ uncertainties that raised a similar question were grouped together to form a summary question. This created a long list of summary questions. The data analysts then conducted a literature search to remove summary questions that have already been answered and were evidence already existed; Pubmed/Cochrane/clinical trial registries were used to search for evidence.

The remaining summary questions were then reviewed by the steering group to determine the contents of the interim prioritisation survey.

### 2.5 Interim prioritisation survey

The second survey was released in October 2021. Respondents of the survey were asked to look at the research questions and vote up to 10 choices they would like to see prioritised. The survey was circulated electronically using the same resources as the first survey. The 24 research questions that received the most votes from the interim survey were included in the list on the final workshop.

### 2.6 Final workshop

A one‐day in‐person workshop involving patient representatives, healthcare professionals, and other stakeholders was held in Manchester in November 2021. The top 24 research questions picked from the interim survey were discussed and ranked to the final top 10 list of research questions. Participants in the final workshop were split into two groups, and each group facilitated by an independent JLA adviser. The first round of small group working involved discussion of each person's top three and lowest three priorities. In the second small group session, the participants ranked all questions in order 1–24. The rankings of the two groups were combined using a simple arithmetic mean, which was also checked using a geometric mean. After an intermission, the two groups were mixed up, to ensure all participants heard a range of perspectives. Each group reviewed the previously combined ranking and adjusted as they felt appropriate. The rankings from the two groups were again combined to provide a new combined ranking. All participants had an opportunity in the final plenary session to suggest adjustments to the order.

## 3 RESULTS

### 3.1 Initial survey

A total of 665 respondents provided 1698 potential research uncertainties. After initial screening of the raw uncertainties, 13 were removed as they were deemed out‐of‐scope by the analysts and steering group. The remaining 1672 uncertainties were then categorised into 13 themes: management/treatment, causes, prognosis, support, health services, symptoms, diagnosis, assessment and investigations, awareness, research, prevention, food and diet, and others. The ‘other’ categories included uncertainties that asked specific questions and did not belong to the other themes.

The data analysts grouped the uncertainties in each category into summary questions. This resulted in 147 summary questions in the initial long list. Following the literature review and discussion with the steering group, 110 summary questions were removed. The long list was then reduced and refined to 37 summary questions to be included in the second survey.

The demographics of the survey respondents can be seen in Table 2 with respondent characteristics summarised as follows: 154 male, 467 female, two identified as non‐binary and 42 preferred not to say. The age range of our respondents ranged from under 18 to over 80 years old, with the median age range being in the 55–64 age group. Respondents were asked to select from a list what symptoms of SATD they experienced, if any, and if they were a health/social care professional, or a researcher in the field. They could respond to as many of these options that applied to them. From the 665 unique responses, most respondents, 551, had a lived experience of a smell and/or taste disorder, 18 cared for someone with a smell and/or taste disorder, 28 were health/ social care professional, 10 were researchers in the field, and some respondents had chosen not to select any of the options.

**TABLE 2:** Initial survey demographics (*n* = 665)

(a) SATD status	*N* (%)
I have no sense of smell (anosmia)	314 (47.2)
I have reduced sense of smell (hyposmia)	170 (25.6)
I have smell distortion	163 (24.5)
I experience smell hallucination	84 (12.6)
I have fluctuating symptoms	116 (17.4)
I have no sense of true taste (ageusia)	174 (26.2)
I have partial loss of true taste (hypogeusia)	171 (25.7)
I experience a distortion of true taste (dysgeusia)	100 (15.0)
I experience a bad smell that comes from within my body	54 (8.1)
I have previously had a smell and/or taste disorder but am recovered	22 (3.3)
I care for/live with/support someone with a smell and/or taste disorder	18 (2.7)
I am responding as a health/socialcare professional	28 (4.2)
I am a researcher in this field	10 (1.5)
I have another reason for responding (please specify)	58 (8.7)
(b) Self‐reported aetiology	
Allergies	7 (1.1)
Born without a sense of smell (congenital anosmia)	29 (4.4)
Chronic rhinosinusitis (with or without nasal polyps)	61 (9.2)
COVID‐19	99 (14.9)
Head injury	69 (10.4)
I do not have a diagnosed reason (idiopathic)	115 (17.3)
Not applicable‐I do not have/am not supporting someone with a smell/taste disorder	119 (17.9)
Parkinson's disease	2 (0.3)
Smell disorder caused by an infection, common cold, flu, etc	99 (14.9)
Other nasal blockages, for example, deviated nasal septum	5 (0.8)
Other (please specify)	113 (17.0)
No response	47 (7.1)
(c) Duration of symptoms	
1–3 years	94 (14.1)
3–5 years	78 (11.7)
5 years	269 (40.5)
Less than 12 months	121 (18.2)
Not applicable	22 (3.3)
Since birth	44 (6.6)
No response	37 (5.6)
(d) Have you (or the person you support) seen a doctor/specialist for smell/taste disorders?	
No	256 (38.5)
NA	22 (3.3)
Yes	350 (52.6)
No response	37 (5.6)
(e) Have you (or the person you support) received any treatment for smell/taste disorders?	
no	424 (63.8)
yes	180 (27.1)
NA	23 (3.5)
No response	38 (5.7)
(f) Race/ethnicity	
White‐British/Irish	440 (66.2)
White‐any other background	128 (19.2)
Asian/Asian British‐Chinese	2 (0.3)
Asian/Asian British‐Indian	11 (1.7)
Asian/AsianBritish‐any other Asian background	3 (0.5)
Black/Black British‐African	1 (0.2)
Black/Black British‐Caribbean	3 (0.5)
Black/Black British‐any other Black background	2 (0.3)
Hispanic/Latino	9 (1.4)
Mixed/Multiple Ethnicity‐any Asian/White	4 (0.6)
Mixed/Multiple Ethnicity‐any Black/White	2 (0.3)
Other‐any other ethnicity not listed	9 (1.4)
Prefer not to say/No response	51 (7.7)
(g) Gender	
Male	154 (23.2)
Female	467 (70.2)
Non‐binary	2 (0.3)
Prefer not to say	42 (6.3)
(h) Age group	
Under 18	5 (0.8)
18–24	19 (2.9)
24–34	44 (6.6)
35–44	41 (6.2)
45–54	105 (15.8)
55–64	183 (27.5)
65–79	204 (30.7)
80+	27 (4.1)
No response	37 (5.6)

### 3.2 Interim prioritisation survey and final work

The steering group reviewed these results to generate a final shortlist of 37 questions that were to be included in the interim survey. A total of 235 people responded to the interim survey and gave their priorities.

The final workshop was conducted on the 19 November 2021 with 20 attendees including two members of JLA to facilitate the discussion, 12 patient participants, and six clinicians. After two rounds of discussion, the groups reached a consensus of the final top 10 research questions with two questions being modified in agreement with all present (Table 3). Many of the summary questions in the top 10 focussed on possible treatments for SATDs.

**TABLE 3:** Ranked priorities

Question	Final rank
How can we further our understanding of the mechanism of disease in the nerve pathways that affect smell and taste disorders, including where parosmia and phantosmia exist.	1
How can medical professionals be better educated in treating smell/taste disorders?	2
Do stem cells have the potential to treat smell and taste disorders?	3
How can regeneration of smell receptors be used to treat smell or taste disorders?	4
What are the mental health consequences of smell/taste disorders and how can these be managed effectively?	5
How can medical technology (e.g., implants) be used to rehabilitate sense of smell/taste?	6
How can the testing and investigations into smell/taste disorders be improved?	7
What role does genetics play in smell/taste disorders?	8
Are there any effective treatments for smell and taste disorders due to COVID‐19 or any other viral infection?	9
What coping strategies help in dealing with smell/taste disorders?	10
How can we determine the underlying mechanism for those smell and taste disorders that have no apparent cause?	11
How can the understanding of the fluctuation and variation of symptoms be improved?	12
What is the prognosis for different smell and taste disorders and what factors affect it?	13
Are smell and taste disorder symptoms affected by hormone changes, such as those associated with pregnancy, periods, or menopause?	14
How can smell and taste disorders resulting from head injury be effectively treated?	15
How can home remedies or alternative therapies (acupuncture, herbal, homeopathic, etc) help with the recovery of taste and smell?	16
How can the safety risk (spoilt food, smoke, etc) that comes with smell and taste disorders be reduced?	17
Do surgical interventions have the potential to improve the management of smell and taste disorders?	18
Are there possible treatments for people who have lost their sense of smell from having Parkinson's disease?	19
Are there any food or vitamins/food supplements that can help with smell and taste?	20
How effective is smell training when a person has had anosmia for many years?	21
How can other senses be used to compensate for loss of smell and taste?	22
How can lifestyle habits (e.g., exercise, mindfulness) help with smell and taste disorders?	23
How can enjoyment of food and drink be improved for people experiencing smell and taste disorders?	24

## 4 DISCUSSION

This study reports the shared research priorities representing the unanswered questions of patients, carers, and healthcare professionals. This was the first formulaic exercise of this kind internationally using the JLA methodology, although we note a previous multistakeholder workshop had been convened in the USA in 2019.^19^ Patients, carers, researchers, and healthcare professionals were actively involved in all stages of the process to ensure that the stakeholder voice remained at the centre of our work. All the original research submissions and the summary questions are available on the JLA website in the final report. The demographics and aetiology of study participants were in keeping with the typical female predominance seen in other studies including our own.^16^, ^20^, ^21^

### 4.1 Limitations

The cohort of the initial survey consisted largely of older, white British/Irish, and female demographic, which reduces the generalisability of the findings, by not encapsulating a more diverse demographic. This demographic could explain how the uncertainty ‘Are smell and taste disorder symptoms affected by hormone changes, such as those associated with pregnancy, periods, or menopause?’ was initially popular. Despite strong arguments being given to include this question, during the final workshop, it was not included in the top 10 list as although it impacts the large majority of our initially survey respondents, it does not impact the overall population of people with SATD.

As the survey was only sent out electronically, it will not have been available to those who do not have access to the Internet, likely to disproportionately affect older generations. It is, however, encouraging that 30.7% (*n* = 204) were aged 65–79 years and 4.1% (*n* = 27) were over 80 years. Throughout all stages of the process, there was limited response from healthcare professionals; nevertheless, the steering group itself was balanced and aimed to ensure proportionate representation of all respondents' research priorities.

Most respondents who have SATDs had seen a doctor/specialist for SATDs (52.6%), but most of them have not received any treatment for it (63.8%). This could explain the large number of uncertainties raised initially related to treatment. This was taken to account during our initial screening process, and uncertainties that merely ask what treatment was available were deemed out of scope as it was not asking a research question.

The large majority of people who responded to the initial survey were people who had lived experience of SATD, while only 28 identified as health and social care professional and 10 were researchers. This resulted in most of the summary questions derived to be removed as they were questions that had already been answered by research (110 out of 147). This highlights an existing lack of communication with patients, members of public, and some professionals regarding treatment and research available for SATDs.

### 4.2 Generalisability

The effects of the COVID‐19 Pandemic were visible in the results of our initial survey, as 15% (*n* = 99) of respondents had a loss of taste or smell following COVID‐19, representing the increasing number of people affected due to the virus. One of the research questions that asked about treatment for smell loss due to COVID‐19 made it to the top 10 list of research questions, reflecting the increasing concern of this issue in the community.

The initial survey generated many very specific uncertainties. However, during the Final Workshop, broader research questions attracted more votes. The top research aim: ‘How can we further our understanding of the mechanism of disease in the nerve pathways that affect smell and taste disorders, including where parosmia and phantosmia exist’. was created as both groups in the workshop agreed that these broader uncertainties could be rephrased to include some of the other more specific uncertainties, thus representing the overarching message in one uncertainty. Despite this, we aimed to ensure a balance between specific and more general questions, to ensure the original submissions were appropriately represented.

## 5 CONCLUSION

This work has generated the top research questions in SATDs and has highlighted the impact SATDs can have in an individual that often goes overlooked by healthcare professionals. Using this information, the research community will be empowered to engage research funders and aim to deliver answers to these questions. FifthSense has now established Research Hubs set up specifically to deal with the emerging themes identified by the JLA PSP and these are now visible on their website (https://www.fifthsense.org.uk/research/).

## AUTHOR CONTRIBUTIONS

Carl M. Philpott was professional lead, member of steering group and reviewed the manuscript. Andreas Espehana, Mairenn Garden served as data analyst and wrote and reviewed the manuscript. Ngan Ta, Nishita Gadi, Kala Kumaresan served as data analysts and reviewed the manuscript. Devina Maru, Lorenzo D. Stafford, Nina Bleasdale and Duncan Boak were members of the steering group and reviewed the manuscript.

## FUNDING INFORMATION

FifthSense funded the James Lind Alliance Priority Setting Partnership.

## CONFLICT OF INTEREST

Some of the authors are currently involved in research relating to some of the research priorities derived from the PSP. An independent representative from the James Lind Alliance was present during each step of the PSP process to ensure any conflicts of interest were declared and bias was minimised.

### PEER REVIEW

The peer review history for this article is available at https://publons.com/publon/10.1111/coa.13985.

## Data Availability

The data from the study are available at the JLA website.
